# Temporal Variations in Convergence Insufficiency Symptomatic Status among University Students before and after COVID-19: A Longitudinal Analysis from 2018 to 2023

**DOI:** 10.3390/life14070800

**Published:** 2024-06-26

**Authors:** Hugo Pena-Verdeal, Veronica Noya-Padin, Jacobo Garcia-Queiruga, Noelia Nores-Palmas, Maria J. Giraldez, Eva Yebra-Pimentel

**Affiliations:** 1 Applied Physics Department (Optometry Area), Facultade de Óptica e Optometría, Universidade de Santiago de Compostela, 15705 Santiago de Compostela, Spain; hugo.pena.verdeal@usc.es (H.P.-V.);; 2 Optometry Group, Instituto de Investigación Sanitaria Santiago de Compostela (IDIS), 15706 Santiago de Compostela, Spain

**Keywords:** convergence insufficiency symptom survey, COVID-19 pandemic education, odds ratio

## Abstract

The present study aimed to assess the symptomatic status of Convergence Insufficiency (CI) in university students from 2018 to 2023 considering the educational environment pre- and post-COVID-19 pandemic confinements. A Convergence Insufficiency Symptom Survey (CISS) was conducted annually from 2018 to 2023, excluding 2020, in an initial group of 217 third-year Optics and Optometry degree university student participants. In the final group (178 participants), the statistical differences in CISS scores between years were analysed, both overall and by questionnaire subgroup, along with associations between CISS diagnostic categories before and after 2020. Significant differences were found between years in the subscale and total score analyses (Kruskal–Wallis, both *p* ≤ 0.049). Pairwise comparisons showed significant differences for the performance subgroup in 2021 vs. 2019 and 2018 (Mann–Whitney, both *p* ≤ 0.004), while in terms of the total score, there was a statistical difference in 2021 vs. 2018 (Mann–Whitney, *p* < 0.001). The distribution analysis indicated a significant difference between groups (Chi, *p* = 0.004), with participants from 2021 or later more likely to exhibit higher CISS scores (OR = 3.47, 95%CI 1.04–8.58). The present study shows significant temporal increments in symptomatic status related to CI among university students from 2018 to 2023, indicating a potential impact of the COVID-19 pandemic educational landscape on these outcomes.

## 1. Introduction

Binocular or accommodative visual anomalies constitute a prevalent set of dysfunctions that can impair the efficiency of the visual system [1,2]. The absence of a proper diagnosis or treatment for accommodative/vergence anomalies may lead to various visual problems, such as amblyopia, diminished binocular function, or stereopsis, potentially resulting in learning disabilities or even behavioural disorders [3,4,5]. Among the most common binocular or accommodative visual anomalies are Convergence Insufficiency (CI) and Accommodative Insufficiency (AI), impacting a population ranging from 1.7% to 43% of individuals over the age of 19 years old [6,7,8]. CI is characterised by difficulty maintaining fusion while focusing on a near target, attributed to the tendency of the eyes to drift outwards. On the other hand, AI affects one’s ability to sustain focus with near vision for extended periods [8]. These disorders often manifest with symptoms such as eyestrain, headaches after short reading periods, visual fatigue, blurred or double vision, sleepiness, difficulty concentrating, and challenges in reading comprehension. Given that symptom assessment is crucial for diagnosing and monitoring those disorders, the Convergence Insufficiency Symptom Survey (CISS) was developed by the Convergence Insufficiency and Reading Study [9,10].

During the year 2020, the global impact of the COVID-19 pandemic led to various lockdowns, stay-at-home orders, and societal restrictions, implemented with varying frequencies and indications across different countries [11]. From 2020 to 2022, the worldwide education sector experienced significant disruptions, affecting student learning and limiting in-person educational activities [12]. Digital methods became the primary mode for educational activities, even as some countries lifted or eased restrictions. Despite these changes, digital processes have become firmly established as a common method of learning [13]. The shift towards digital learning methods precipitated by the COVID-19 pandemic has raised concerns regarding its effects on general and visual health [14,15,16]. Increased screen time, prolonged exposure to digital devices, and changes in study environments may exacerbate existing visual anomalies or contribute to the emergence of new issues among university students. Studies have indicated a potential link between prolonged screen exposure and symptoms such as eyestrain, dry eyes, and visual fatigue, which are commonly associated with conditions like CI and AI [17]. Moreover, the prevalence of digital eyestrain and related symptoms has been observed to increase among individuals engaging in prolonged screen-based activities, such as reading from electronic devices or attending virtual lectures [17]. Understanding the specific challenges posed by digital learning environments is crucial for addressing the evolving needs of students and implementing targeted interventions to promote visual health [18]. Studying these effects and their implications for long-term visual health is essential for developing strategies to mitigate the potential risks and promote optimal visual well-being in digital learning environments. This study aims to compare the CISS symptomatic status among university students between 2018 and 2023 considering the evolving educational landscape, influenced by the COVID-19 pandemic.

## 2. Materials and Methods

### 2.1. Sample and Study Desing

A CISS assessment was conducted annually from 2018 to 2023 (excluding the year 2020) during the months of October and November. Each year, the investigation focused on university students who were in their third year of the Optics and Optometry degree program. Participants were included if they were within the age range of 19 to 26 years, had no prior diagnoses of ocular pathology, previous binocular or accommodative diagnosed disorders, or dry eye disease, and were not under treatment or taking medications that may have influenced the symptomatic results of the test [19,20,21]. From an initial recruited group of 217 participants, the final analysis group was composed of 178 participants, divided into 5 analysis groups (one per year: 2018, 2019, 2021, 2022, and 2023), who completed the questionnaire and met the inclusion and exclusion criteria. In all cases, questionaries were administrated by one qualified optometrist with several years of experience employing this tool. The questionnaires were evaluated each year by a second optometrist who was masked and not aware of the results from other years.

All the participants gave their written informed consent to be included in the study, and all procedures were followed in accordance with the Declaration of Helsinki. Recorded data were alphanumerically masked prior to the analysis. The study protocol for the initial regular recording of data was approved by the Galicia Clinical Research Ethics Committee (CEIC-G: 2013/360), and the retrospective and prospective protocols for data analysis were approved by the Bioethics Committee of the University of Santiago de Compostela (USC-08/2021).

### 2.2. The Convergence Insufficiency Symptom Survey (CISS)

The CISS questionnaire is a 15-item questionnaire [10,22,23] (Table 1). For each questionnaire item, the patient chooses one of five possible responses (never, infrequently, sometimes, often, and always). Each answer is scored from 0 to 4, with 4 indicating the highest frequency of the symptom (always). The overall CISS score is the sum of the scores of all 15 items, ranging from 0 (completely asymptomatic) to 60 (completely symptomatic) [22,23]. From the total score, participants may be classified as symptomatic if the CISS average score is 21 or greater [22]. Additionally, the questions can be divided into two subscales: a subscale related to visual performance (items 4, 5, 6, 9, 14, 15) and a subscale related to ocular symptoms themselves (items 1, 2, 3, 7, 8, 10, 11, 12, 13) [23].

### 2.3. Statistical Analysis

Statistical Package for the Social Sciences statistical software v.25.0 for Windows (SPSS Inc., Armonk, NY, USA) was used for data analysis. Significance was set at *p* ≤ 0.05 for all the statistical tests [24]. Before analysis, the normal distribution of the data was checked using the Kolmogorov–Smirnov test. The CISS score values for both subscales and the total scores showed a non-normal distribution (Kolmogorov–Smirnov test: all *p* ≤ 0.001) [25].

As the first analysis, differences in the CISS scores between years, both total score and by the subscales of the tests, were assessed using the Kruskal–Wallis test, while the Mann–Whitney test was used to detect significant pairwise differences; to avoid type I errors arising from multiple comparisons in the axis values, statistical significance for the Mann–Whitney test was divided by the number of comparisons performed to give a *p* ≤ 0.005 (Bonferroni adjustment) [26,27].

In a second analysis, participants were categorised both based on when the test was performed (prior to 2020–2018–2019 [Year ≤ 2019] or after 2020–2021–2023 [Year ≥ 2021]) and whether the CISS average score was lower or greater than 21 points (CISS ≤ 21 and CISS ≥ 21 respectively). Due to the categorical nature of the data, a chi-squared design was used to compare the outcomes of the risk factors studied across different CISS categories [24]. Odds ratios (ORs), along with 95% confidence intervals (CIs), were estimated to assess the magnitude of the association between participants studied previously and before the year 2020 with the CISS category [24].

## 3. Results

### 3.1. Differences between Scores by Years

There was a statistically significant difference between years in both the subscale analysis and the total score analysis (Kruskal–Wallis, all *p* ≤ 0.049, Table 2). In the pairwise analysis of the visual performance subscale, a statistical difference was observed only in the 2018 vs. 2021 and 2019 vs. 2021 comparisons (Mann–Whitney, both *p* ≤ 0.004, Table 2), while all the other year-to-year comparisons showed no statistical difference (Mann–Whitney, all *p* ≥ 0.062, Table 2). The pairwise analysis of the eye ocular symptom subscale revealed no statistical differences between years in any of the comparisons (Mann–Whitney, all *p* ≥ 0.006, Table 2). In the pairwise analysis of the total score, there was a statistical difference in the 2018 vs. 2021 comparison (Mann–Whitney, *p* < 0.001, Table 2), whereas all the other year-to-year comparisons showed no statistical difference (Mann–Whitney, all *p* ≥ 0.009, Table 2).

### 3.2. Associations between Year Group and CISS Score

There was a statistically significant association in the distribution between groups (Chi, *p* = 0.004, Table 3). Compared to the Year ≤ 2019 group, the Year ≥ 2021 participants were more likely to have a CISS score ≤ 21 (OR 3.47, 95% CI 1.04–8.58)

## 4. Discussion

The prevalence of binocular and accommodative visual anomalies, such as CI and AI, raises concerns due to their potential impacts on the visual system’s efficiency. Untreated anomalies may lead to various visual issues, including amblyopia and learning disabilities [3,4,5]. CI and AI, common in individuals aged 19 years old and older, manifest with symptoms like eyestrain and difficulty concentrating [6,7,8]. The CISS plays a crucial role in assessing and monitoring CI [12,22,23]. Among global disruptions from the COVID-19 pandemic [11], the present study explores the symptomatic status of university students from 2018 to 2023, considering the evolving educational landscape, influenced by the pandemic. The CISS questionnaire has been validated as a reliable tool for binocular or accommodative problem assessment [19,28] and has even been used in prevalence screening studies as the main tool [29]. While previous studies evaluated the CISS scores in university students in the pre-COVID period and the period of confinement due to the pandemic [30], to the authors’ knowledge, no studies have compared the pre-COVID period with long-term values from before the end of confinement measures.

Statistical analyses revealed significant differences between years, both in the subgroup and total score analyses. Pairwise comparisons indicated significant differences only in specific year-to-year instances, emphasising the impact of the evolving educational landscape on symptomatic status. Notably, the 2018 vs. 2021 comparison exhibited a significant difference in the total score, suggesting a potential influence of pandemic-related educational shifts. Additionally, the distribution analysis revealed a significant difference between groups, with participants from 2019 or earlier being more likely to exhibit lower CISS scores, indicative of reduced symptomatic presentation. These results are in concordance with the trend obtained in previous studies, with an increase in the number of CISS symptomatic cases among university students in the pre-COVID period and in the period of confinement due to the pandemic [30]. During confinement, due to the increase in the use of digital devices, there was a notable increase in CI [31]. Regarding confinement or the post-confinement period, some studies have reported an increase in CISS scores in students between 10 and 17 years old who attended full-time or hybrid virtual education [32] and detrimental to abnormal binocular vergence and accommodation parameters in a similar population who attended long-term online classes [33]; both results are in concordance with the increase in an actual highly digitalised post-confinement educational environment.

Understanding the impact of binocular and accommodative visual anomalies on the overall well-being of university students underscores the importance of implementing effective interventions and preventive measures. From the present results, it could be hypothesised that addressing these visual challenges through targeted interventions and proactive strategies may promote general and visual health in educational settings [18]. Interventions may include education on proper visual hygiene practices and ergonomic adjustments to mitigate the adverse effects of prolonged digital device use [34]. Moreover, educational environments may encourage regular breaks from screen time and promote outdoor activities that can contribute to reducing visual discomfort and preventing the onset or progression of visual anomalies [35]. Educators and healthcare professionals play a relevant role in protecting the visual health and academic success of the student population [36].

To propose a continuous monitoring approach for interventions across various educational levels (primary, secondary, and university education), a multifaceted strategy could be employed. This would involve regular vision screenings and assessments using tools like the CISS across different educational stages, creating a longitudinal database to track the visual health of students over time. Additionally, integrating vision health education into school curriculums and providing training for educators to recognise signs of visual stress can help in early identification and intervention. The use of digital platforms and mobile applications for self-monitoring by students and real-time reporting to healthcare providers could further enhance continuous monitoring. Such comprehensive strategies are essential to address the increasing prevalence of binocular dysfunctions and the extensive use of electronic devices from early childhood through to working age.

The primary strength of the present study lies in the consistency of the measurements, as they were uniformly taken under the same conditions at the same point in the academic year among students at an exactly analogous stage of educational progression and with similar ages and habits. While the results may align with existing findings on the visual effects of increased screen time and digital learning, this study contributes by providing a longitudinal analysis spanning multiple years, including both the pre- and post-pandemic periods. There are no previous studies that provide data from several years before the confinement on the present studied issue, nor that include a long-term post-confinement study with such precise timing. This study offers unique insights into the temporal changes in CI symptomatology over a span of five years, excluding 2020, which allows for a detailed analysis of the trends before, during, and after pandemic-related educational shifts.

However, the results may be limited by the individual sample size for each year, potentially limiting the generalisability of the findings to broader populations of university students. In addition, the educational and geographical participant demographics may restrict the applicability of the results to other student populations. Furthermore, although the investigation focuses on symptomatology, a secondary confirmation of the theory through clinical signs (such as the measurement of phorias, near point of convergence, etc.) would have reinforced the proposed hypotheses. The CISS is a well-validated tool for assessing the symptoms associated with CI and is widely used for screening purposes; however, this test alone is not sufficient for a definitive diagnosis of CI. Investigating the underlying mechanisms linking digital device use to the development of binocular and accommodative visual anomalies could provide valuable insights for the development of targeted interventions and preventive strategies. Longitudinal studies assessing the long-term effects of digital learning modalities on visual health are essential for understanding the trajectory of visual anomalies over time and informing evidence-based recommendations to optimise visual well-being in educational settings.

## 5. Conclusions

In conclusion, the present study provides insights into the evolving symptomatic status of university students regarding binocular and accommodative visual anomalies, considering the impact of the COVID-19 pandemic on the educational environment. This adds significant value to the existing body of research by offering a comprehensive temporal perspective. The findings underscore the need for ongoing monitoring of the interventions performed to address visual anomalies in the context of educational methods. Proposing a framework for continuous monitoring, it is recommended to integrate regular vision screenings, educational programs on visual health, and the use of digital tools for real-time monitoring across all educational levels. Such measures are vital to address the increasing challenges posed by digital device use and to ensure the visual well-being of students from primary education through to university.

## Figures and Tables

**Table 1:** Convergence Insufficiency Symptom Survey questions.

Question	Answers				
1. Do your eyes feel tired when reading or doing close work?	Never	Infrequently (not very often)	Sometimes	Fairly often	Always
2. Do your eyes feel uncomfortable when reading or doing close work?	Never	Infrequently (not very often)	Sometimes	Fairly often	Always
3. Do you have headaches when reading or doing close work?	Never	Infrequently (not very often)	Sometimes	Fairly often	Always
4. Do you feel sleepy when reading or doing close work?	Never	Infrequently (not very often)	Sometimes	Fairly often	Always
5. Do you lose concentration when reading or doing close work?	Never	Infrequently (not very often)	Sometimes	Fairly often	Always
6. Do you have trouble remembering what you have read?	Never	Infrequently (not very often)	Sometimes	Fairly often	Always
7. Do you have double vision when reading or doing close work?	Never	Infrequently (not very often)	Sometimes	Fairly often	Always
8. Do you see the words move, jump, swim, or appear to float on the page when reading or doing close work?	Never	Infrequently (not very often)	Sometimes	Fairly often	Always
9. Do you feel like you read slowly?	Never	Infrequently (not very often)	Sometimes	Fairly often	Always
10. Do your eyes ever hurt when reading or doing close work?	Never	Infrequently (not very often)	Sometimes	Fairly often	Always
11. Do your eyes ever feel sore when reading or doing close work?	Never	Infrequently (not very often)	Sometimes	Fairly often	Always
12. Do you feel a “pulling” feeling around your eyes when reading or doing close work?	Never	Infrequently (not very often)	Sometimes	Fairly often	Always
13. Do you notice the words blurring or coming in and out of focus when reading or doing close work?	Never	Infrequently (not very often)	Sometimes	Fairly often	Always
14. Do you lose your place while reading or doing close work?	Never	Infrequently (not very often)	Sometimes	Fairly often	Always
15. Do you have to re-read the same line of words when reading?	Never	Infrequently (not very often)	Sometimes	Fairly often	Always

**Table 2:** Descriptive statistics and analysis of differences (Kruskal–Wallis test) in the total and subscales scores of each year.

		Year	n	Median	IQR	Minimum	Maximum	*p*
Subscales	Visual performance	2018	38	4.5	5.0	0	16	0.012
		2019	46	5.0	5.0	0	13	
		2021	37	7.0	4.5	1	14	
		2022	28	6.0	3.0	1	17	
		2023	29	4.0	4.5	0	15	
	Ocular symptoms	2018	38	5.0	6.3	0	16	0.049
		2019	46	7.5	5.2	0	24	
		2021	37	9.0	10.0	0	23	
		2022	28	7.0	9.8	0	24	
		2023	29	9.0	8.0	0	22	
Total score		2018	38	11.0	9.0	0	30	0.011
		2019	46	14.0	9.0	0	37	
		2021	37	17.0	10.5	3	34	
		2022	28	12.5	11.2	2	34	
		2023	29	14.0	17.0	3	32	

IQR = interquartile range.

**Table 3:** Distribution of the Year ≤ 2019 and Year ≥ 2021 participants based on the CISS symptomatic status.

	CISS ≤ 21	CISS ≥ 21	Total	OR (95% CI)	*p*
Year ≤ 2019	76	7	83	3.47 (1.04–8.58)	0.004
Year ≥ 2021	72	23	95		
	148	30	178		

OR = odds ratio. CI = confidence interval. CISS = Convergence Insufficiency Symptom Survey.

## Data Availability

The data are unavailable due to privacy restrictions.

## References

[1] Yekta A., Khabazkhoob M., Hashemi H., Ostadimoghaddam H., Ghasemi-Moghaddam S., Heravian J., Doostdar A., Nabovati P. (2017). Binocular and Accommodative Characteristics in a Normal Population. Strabismus.

[2] Scheiman M., Wick B., Wilkins L.W. (2017). Clinical Management of Binocular Vision: Heterophoric, Accommodative, and Eye Movement Disorders.

[3] Granet D.B., Gomi C.F., Ventura R., Miller-Scholte A. (2005). The relationship between convergence insufficiency and ADHD. Strabismus.

[4] Brautaset R., Wahlberg M., Abdi S., Pansell T. (2008). Accommodation insufficiency in children: Are exercises better than reading glasses?. Strabismus.

[5] Scheiman M., Cotter S., Kulp M.T., Mitchell G.L., Cooper J., Gallaway M., Hopkins K.B., Bartuccio M., Chung I., Convergence Insufficiency Treatment Trial Study Group (2011). Treatment of accommodative dysfunction in children: Results from a randomized clinical trial. Optom. Vis. Sci..

[6] Ghadban R., Martinez J.M., Diehl N.N., Mohney B.G. (2015). The incidence and clinical characteristics of adult-onset convergence insufficiency. Ophthalmology.

[7] Cooper J., Jamal N. (2012). Convergence insufficiency—A major review. Optometry.

[8] Nunes A.F., Monteiro P.M.L., Ferreira F.B.P., Nunes A.S. (2019). Convergence insufficiency and accommodative insufficiency in children. BMC Ophthalmol..

[9] Borsting E., Rouse M.W., De Land P.N. (1999). Prospective comparison of convergence insufficiency and normal binocular children on CIRS symptom surveys. Convergence Insufficiency and Reading Study (CIRS) group. Optom. Vis. Sci..

[10] Lavrich J.B., Hamburger J.L., Lee K.E., Thuma T.B.T., Omega M.L., Zhang Q.E., Gunton K.B. (2023). Creating consistency in the diagnosis of convergence insufficiency: Screening methods. J. AAPOS.

[11] Alfano V., Ercolano S. (2020). The Efficacy of Lockdown Against COVID-19: A Cross-Country Panel Analysis. Appl. Health Econ. Health Policy.

[12] Amer F., López T., Gil-Conesa M., Carlos S., Ariño A., Carmona-Torre F., Martínez-González M., Fernandez-Montero A. (2023). Association between COVID-19 and outstanding academic performance at a Spanish university. Arch. Public Health.

[13] Drossard S., Hartl A. (2024). Development and implementation of digital peer mentoring in small groups for first-year medical students. GMS J. Med. Educ..

[14] Garcia-Queiruga J., Pena-Verdeal H., Sabucedo-Villamarin B., Giraldez M.J., Garcia-Resua C., Yebra-Pimentel E. (2023). Meibomian gland secretion quality association with ocular parameters in university students during COVID-19 restrictions. Int. Ophthalmol..

[15] Chang C.C., Hsieh K.Y., Hsu S.T., Wang Y.Y., Chou F.H., Huang J.J. (2024). Understanding the mental health impacts of biological disasters: Lessons from Taiwan’s experience with COVID-19. J. Formos. Med. Assoc..

[16] Kang T., Lee Y., Kang M. (2024). Impact of COVID-19 on healthcare utilization among chronic disease patients in South Korea. Prev. Med. Rep..

[17] AlQarni A.M., AlAbdulKader A.M., Alghamdi A.N., Altayeb J., Jabaan R., Assaf L., Alanazi R.A. (2023). Prevalence of Digital Eye Strain Among University Students and Its Association with Virtual Learning During the COVID-19 Pandemic. Clin. Ophthalmol..

[18] Oszczedlowski P., Raczkiewicz P., Wiesyk P., Brzuszkiewicz K., Rapa M., Matysik-Wozniak A., Zielinski G., Onyszkiewicz M., Rekas K.M., Makosz I. (2023). The Incidence and Severity of Myopia in the Population of Medical Students and Its Dependence on Various Demographic Factors and Vision Hygiene Habits. Int. J. Environ. Res. Public Health.

[19] Pang Y., Gabriel H., Tan Q.Q. (2023). Convergence insufficiency symptom survey: A tool to evaluate convergence excess in young adults. Ophthalmic Physiol. Opt..

[20] Kothari M., Modak M., Khan H., Jahan S., Solanki M., Rathod V. (2020). Convergence excess consecutive esotropia associated with 0.01% atropine eye drops usage in patients operated for intermittent exotropia. Indian J. Ophthalmol..

[21] Pena-Verdeal H., Garcia-Queiruga J., Sabucedo-Villamarin B., Giraldez M.J., Garcia-Resua C., Yebra-Pimentel E. (2023). Capability of the Inter-Eye Differences in Osmolarity, Break-Up Time and Corneal Staining on the Diagnostic of Dry Eye. Ocul. Immunol. Inflamm..

[22] Bolding M.S., Lahti A.C., Gawne T.J., Hopkins K.B., Gurler D., Gamlin P.D. (2012). Ocular convergence deficits in schizophrenia. Front. Psychiatry.

[23] Rouse M.W., Borsting E.J., Mitchell G.L., Scheiman M., Cotter S.A., Cooper J., Kulp M.T., London R., Wensveen J., Convergence Insufficiency Treatment Trial G. (2004). Validity and reliability of the revised convergence insufficiency symptom survey in adults. Ophthalmic Physiol. Opt..

[24] Armstrong R.A., Davies L.N., Dunne M.C., Gilmartin B. (2011). Statistical guidelines for clinical studies of human vision. Ophthalmic Physiol. Opt..

[25] Armstrong R.A. (2017). Recommendations for analysis of repeated-measures designs: Testing and correcting for sphericity and use of manova and mixed model analysis. Ophthalmic Physiol. Opt..

[26] Armstrong R.A. (2014). When to use the Bonferroni correction. Ophthalmic Physiol. Opt..

[27] Noya-Padin V., Nores-Palmas N., Giraldez M.J., Yebra-Pimentel E., Pena-Verdeal H. (2023). Comparison Between Ocular Biometric Parameters and Intraocular Pressure With and Without Contact Lenses. Eye Contact Lens.

[28] Chen A.M., Borsting E.J. (2023). Near work symptoms and measures of accommodation in children. Clin. Exp. Optom..

[29] Junghans B.M., Azizoglu S., Crewther S.G. (2020). Unexpectedly high prevalence of asthenopia in Australian school children identified by the CISS survey tool. BMC Ophthalmol..

[30] Nunes A.F., Leitao M.A., Nunes A.S., Monteiro P.L. (2023). Eye discomfort at close work in Portuguese university students: A comparative analysis between the pre-COVID and confinement period. Work.

[31] Mon-Lopez D., Bernardez-Vilaboa R., Fernandez-Balbuena A.A., Sillero-Quintana M. (2020). The Influence of COVID-19 Isolation on Physical Activity Habits and Its Relationship with Convergence Insufficiency. Int. J. Environ. Res. Public Health.

[32] Hamburger J.L., Lavrich J.B., Rusakevich A.M., Leibowitz J.A., Zhitnitsky M.D., Zhang Q., Makkena A.C., Liu C.K., Oh G.J., Sharpe J.E. (2022). The visual consequences of virtual school: Acute eye symptoms in healthy children. J. AAPOS.

[33] Mohan A., Sen P., Shah C., Datt K., Jain E. (2021). Binocular Accommodation and Vergence Dysfunction in Children Attending Online Classes during the COVID-19 Pandemic: Digital Eye Strain in Kids (DESK) Study-2. J. Pediatr. Ophthalmol. Strabismus.

[34] Biswas S., El Kareh A., Qureshi M., Lee D.M.X., Sun C.H., Lam J.S.H., Saw S.M., Najjar R.P. (2024). The influence of the environment and lifestyle on myopia. J. Physiol. Anthropol..

[35] Devi K.A., Singh S.K. (2023). The hazards of excessive screen time: Impacts on physical health, mental health, and overall well-being. J. Educ. Health Promot..

[36] Langlois S., da Silva Souza C.M., Xyrichis A., Baser Kolcu M.I., Lising D., Najjar G., Khalili H. (2024). Evolving global responses to the pandemic: Sustaining interprofessional education and collaborative practice. J. Interprof. Care.

